# 4,4′-Difluoro-2,2′-[imidazolidine-1,3-diylbis(methyl­ene)]diphenol

**DOI:** 10.1107/S1600536812040329

**Published:** 2012-09-29

**Authors:** Augusto Rivera, Luz Stella Nerio, Jaime Ríos-Motta, Monika Kučeráková, Michal Dušek

**Affiliations:** a Universidad Nacional de Colombia, Sede Bogotá, Facultad de Ciencias, Departamento de Química, Cra 30 No.45-03, Bogotá, Código Postal 111321, Colombia; b Institute of Physics ASCR, v.v.i., Na Slovance 2, 182 21 Praha 8, Czech Republic

## Abstract

In the title compound, C_17_H_18_F_2_N_2_O_2_, the imidazolidine ring system exists in a twist conformation. The mean plane through this ring system forms dihedral angles of 80.8 (8)° and 66.2 (13)°, with the benzene rings. The dihedral angle between the benzene rings is 52.0 (14)°. Two intra­molecular O—H⋯N hydrogen bonds each generate *S*(6) ring motifs. In the crystal, weak C—H⋯O hydrogen bonds form dimers, which are connected by further C—H⋯O inter­actions.

## Related literature

For related structures, see: Rivera *et al.* (2011 ▶, 2012 ▶). For the preparation of the title compound, see: Rivera *et al.* (1993 ▶). For standard bond lengths, see: Allen *et al.* (1987 ▶). For ring conformations, see Cremer & Pople (1975 ▶). For hydrogen-bond graph-set nomenclature, see: Bernstein *et al.* (1995 ▶). For the involvement of organo halides in hydrogen bonds, see: Rathore *et al.* (2011 ▶); Steiner (2002 ▶); Chopra & Guru Row (2005 ▶). For the extinction correction used, see: Becker & Coppens (1974 ▶). 
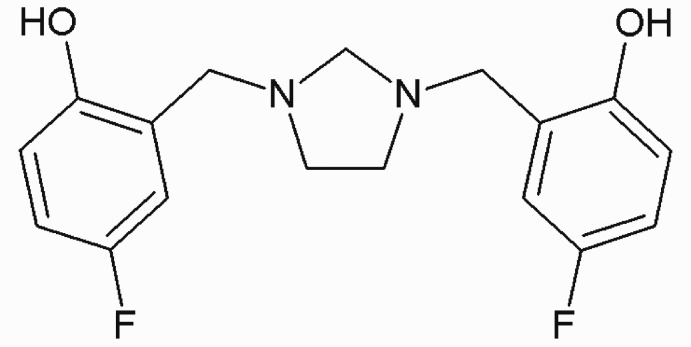


## Experimental

### 

#### Crystal data

C_17_H_18_F_2_N_2_O_2_
*M* *_r_* = 320.3
Monoclinic, 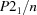

*a* = 9.5952 (2) Å
*b* = 9.7018 (2) Å
*c* = 16.2065 (3) Å
β = 99.4807 (17)°
*V* = 1488.07 (5) Å^3^
*Z* = 4
Cu *K*α radiation
μ = 0.94 mm^−1^
*T* = 120 K
0.35 × 0.22 × 0.21 mm

#### Data collection

Agilent Xcalibur (Atlas, Gemini ultra) diffractometer
Absorption correction: multi-scan (*CrysAlis PRO*; Agilent, 2010 ▶) *T*_min_ = 0.123, *T*_max_ = 1
35554 measured reflections
2669 independent reflections
2452 reflections with *I* > 3σ(*I*)
*R*_int_ = 0.025

#### Refinement

*R*[*F* ^2^ > 2σ(*F* ^2^)] = 0.031
*wR*(*F* ^2^) = 0.108
*S* = 2.21
2669 reflections
215 parameters
H atoms treated by a mixture of independent and constrained refinement
Δρ_max_ = 0.19 e Å^−3^
Δρ_min_ = −0.17 e Å^−3^

### 

Data collection: *CrysAlis PRO* (Agilent, 2010 ▶); cell refinement: *CrysAlis PRO*; data reduction: *CrysAlis PRO*; program(s) used to solve structure: *SUPERFLIP* (Palatinus & Chapuis, 2007 ▶); program(s) used to refine structure: *JANA2006* (Petříček *et al.*, 2006 ▶); molecular graphics: *DIAMOND* (Brandenburg & Putz, 2005 ▶); software used to prepare material for publication: *JANA2006*.

## Supplementary Material

Crystal structure: contains datablock(s) global, I. DOI: 10.1107/S1600536812040329/sj5264sup1.cif

Structure factors: contains datablock(s) I. DOI: 10.1107/S1600536812040329/sj5264Isup2.hkl

Supplementary material file. DOI: 10.1107/S1600536812040329/sj5264Isup3.cml

Additional supplementary materials:  crystallographic information; 3D view; checkCIF report

## Figures and Tables

**Table 1:** Hydrogen-bond geometry (Å, °)

*D*—H⋯*A*	*D*—H	H⋯*A*	*D*⋯*A*	*D*—H⋯*A*
O1—H1⋯N2	0.937 (16)	1.756 (16)	2.6413 (12)	156.2 (14)
O2—H2⋯N1	0.903 (16)	1.821 (15)	2.6579 (12)	153.2 (13)
C11—H1*c*11⋯O1^i^	0.96	2.44	3.4001 (13)	174.43
C17—H2*c*17⋯O1^ii^	0.96	2.55	3.4837 (13)	166

Symmetry codes: (i) 
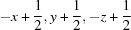
; (ii) 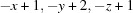.

**Fig. 1.**
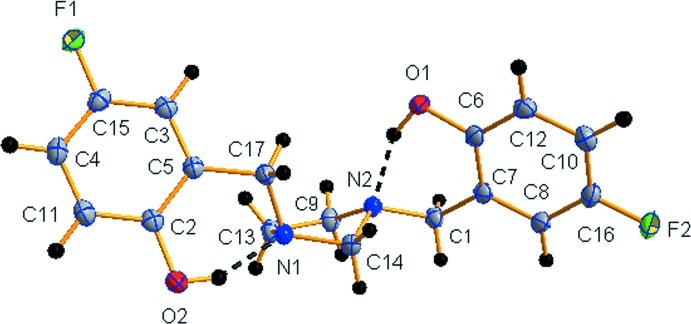
A perspective view of the title compound, showing the atom-numbering scheme. Displacement ellipsoids are drawn at the 50% probability level and H atoms are shown as small spheres of arbitrary radii. Intramolecular hydrogen bonds are drawn as dashed lines.

## supplementary crystallographic information

### Comment

Among the various types of intermolecular interactions, the hydrogen bond is, without doubt, the most important one. Organic halide compounds have attracted much attention due to the role of weak intermolecular C—H···*X* interactions in supramolecular assembly (Rathore *et al.* 2011, Steiner 2002). In recent literature, the importance of interactions involving fluorine as possible tools in crystal engineering has been explored in greater detail (Chopra & Guru Row, 2005). With the purpose to understand its effects in Mannich bases, we turn our attention to title compound (I) because fluorine is also able to form non-classical intermolecular C–H···F hydrogen bonds. In this study, we describe the crystal structure of the title compound, 4,4'-difluoro-2,2'-[imidazolidine-1,3-diylbis(methylene)]diphenol.

The molecular structure and atom-numbering scheme for (I) are shown in Fig. 1. The bond lengths (Allen *et al.*, 1987) and angles of (I) are within normal ranges and are comparable to those related structures (Rivera *et al.*, 2011, 2012). As observed in related structures (Rivera *et al.*, 2011, 2012). The imidazoline ring adopts a twist conformation, Q2 = 0.4008 (13) Å and φ2 = 51.81 (18)° (Cremer & Pople, 1975), with a twist about the N2 —C14 bond. In order to reduce steric congestion, the benzene rings have different orientations with respect to the central imidazolidine ring. Thus, one *p*-fluoro-substituted benzene ring (C1/C2/C5/C10/C6/C17)is approximately orthogonal to the mean plane of the imidazolidine ring defined by N1, C13 and C9, making a dihedral angle of 80.82 (79)°, whereas the other ring (C3/C4/C7/C13/C16/C12) forms a dihedral angle of 66.18 (130)°. The dihedral angle between the benzene rings is 52.04 (136)°. There are two intramolecular hydrogen bonds between the phenolic hydroxyl groups and the nitrogen atoms with graph-set motif S(6) (Bernstein *et al.*, 1995).

The results demonstrate, that not only the packing in (I) is governed by weak C—H···O hydrogen bonds (Table 1), resulting in a hydrogen bonded dimer, which is connected by further C—H···O interactions, but also that fluorine does not participate in any intermolecular interactions. Similarly, in *ortho*-F and *ortho*-Cl substituted analogs (Rivera *et al.*, 2012, 2011), the halogen fails to participate in any non-bonded interaction.

### Experimental

For the originally reported synthesis of the title compound, see: Rivera *et al.* (1993). Crystals suitable for X-ray diffraction were obtained from chloroform with a few drops of MeOH upon slow evaporation of the solvents over 3 days at room temperature.

### Refinement

All hydrogen atoms were discernible in difference Fourier maps and could be refined to reasonable geometry. According to common practice H atoms bonded C atoms were kept in ideal positions with C—H distance 0.96 Å during the refinement. The hydroxyl H atoms were found in difference Fourier maps and their coordinates were refined freely. All H atoms were refined with displacement displacement coefficients U*iso*(H) set to 1.5U*eq*(C, O) for methyl and hydroxyl groups and to to 1.2U*eq*(C) for the CH— and CH_2_— groups.

### Crystal data

**Table:**

C_17_H_18_F_2_N_2_O_2_	*F*(000) = 672
*M**_r_* = 320.3	*D*_x_ = 1.429 Mg m^−^^3^
Monoclinic, *P*2_1_/*n*	Cu *K*α radiation, λ = 1.5418 Å
Hall symbol: -P 2yabc	Cell parameters from 21321 reflections
*a* = 9.5952 (2) Å	θ = 4.6–67.0°
*b* = 9.7018 (2) Å	µ = 0.94 mm^−^^1^
*c* = 16.2065 (3) Å	*T* = 120 K
β = 99.4807 (17)°	Polygon shape, white
*V* = 1488.07 (5) Å^3^	0.35 × 0.22 × 0.21 mm
*Z* = 4	

### Data collection

**Table:**

Agilent Xcalibur (Atlas, Gemini ultra) diffractometer	2669 independent reflections
Radiation source: Enhance Ultra (Cu) X-ray Source	2452 reflections with *I* > 3σ(*I*)
Mirror monochromator	*R*_int_ = 0.025
Detector resolution: 10.3784 pixels mm^-1^	θ_max_ = 67.1°, θ_min_ = 5.0°
ω scans	*h* = −11→11
Absorption correction: multi-scan (*CrysAlis PRO*; Agilent, 2010)	*k* = −11→11
*T*_min_ = 0.123, *T*_max_ = 1	*l* = −19→19
35554 measured reflections	

### Refinement

**Table:**

Refinement on *F*^2^	H atoms treated by a mixture of independent and constrained refinement
*R*[*F*^2^ > 2σ(*F*^2^)] = 0.031	Weighting scheme based on measured s.u.'s *w* = 1/(σ^2^(*I*) + 0.0016*I*^2^)
*wR*(*F*^2^) = 0.108	(Δ/σ)_max_ = 0.009
*S* = 2.21	Δρ_max_ = 0.19 e Å^−^^3^
2669 reflections	Δρ_min_ = −0.17 e Å^−^^3^
215 parameters	Extinction correction: B-C type 1 Gaussian isotropic (Becker & Coppens, 1974)
0 restraints	Extinction coefficient: 900 (400)
66 constraints	

### Special details

**Table:**

Experimental. CrysAlisPro, Agilent, 2010Empirical absorption correction using spherical harmonics, implemented in SCALE3 ABSPACK scaling algorithm.
Refinement. The refinement was carried out against all reflections. The conventional *R*-factor is always based on *F*. The goodness of fit as well as the weighted *R*-factor are based on *F* and *F*^2^ for refinement carried out on *F* and *F*^2^, respectively. The threshold expression is used only for calculating *R*-factors *etc*. and it is not relevant to the choice of reflections for refinement.The program used for refinement, Jana2006, uses the weighting scheme based on the experimental expectations, see _refine_ls_weighting_details, that does not force *S* to be one. Therefore the values of *S* are usually larger than the ones from the *SHELX* program.

### Fractional atomic coordinates and isotropic or equivalent isotropic displacement parameters (Å^2^)

**Table:**

	*x*	*y*	*z*	*U*_iso_*/*U*_eq_	
F1	0.58488 (7)	0.98624 (8)	0.11639 (4)	0.0290 (2)	
F2	0.02275 (8)	0.97367 (8)	0.76918 (5)	0.0339 (3)	
O1	0.37960 (8)	0.95183 (9)	0.53830 (5)	0.0225 (3)	
O2	0.14871 (8)	1.28848 (8)	0.20259 (5)	0.0238 (3)	
N1	0.25088 (9)	1.23815 (10)	0.36231 (6)	0.0205 (3)	
N2	0.32537 (9)	1.21463 (10)	0.50457 (5)	0.0193 (3)	
C1	0.30030 (12)	1.22156 (12)	0.59151 (7)	0.0211 (3)	
C2	0.25940 (11)	1.21368 (11)	0.18359 (7)	0.0203 (3)	
C3	0.45165 (11)	1.05403 (12)	0.21953 (7)	0.0210 (3)	
C4	0.39919 (12)	1.14642 (12)	0.07918 (7)	0.0245 (3)	
C5	0.33984 (11)	1.12752 (12)	0.24310 (6)	0.0195 (3)	
C6	0.28663 (11)	0.96039 (12)	0.59366 (7)	0.0196 (3)	
C7	0.24230 (11)	1.08848 (12)	0.62013 (6)	0.0189 (3)	
C8	0.15072 (11)	1.09088 (12)	0.67872 (7)	0.0217 (3)	
C9	0.40565 (12)	1.33344 (13)	0.48144 (7)	0.0248 (3)	
C10	0.14906 (12)	0.84193 (13)	0.68378 (7)	0.0239 (3)	
C11	0.29049 (12)	1.22389 (12)	0.10290 (7)	0.0236 (3)	
C12	0.23894 (11)	0.83887 (12)	0.62461 (7)	0.0222 (3)	
C13	0.36823 (12)	1.33886 (12)	0.38570 (7)	0.0240 (4)	
C14	0.19724 (11)	1.21598 (12)	0.44037 (6)	0.0206 (3)	
C15	0.47692 (11)	1.06297 (12)	0.13855 (7)	0.0223 (3)	
C16	0.10847 (12)	0.96840 (13)	0.70989 (7)	0.0241 (4)	
C17	0.30169 (11)	1.10785 (12)	0.32918 (7)	0.0211 (3)	
H1c1	0.235801	1.295343	0.597024	0.0253*	
H2c1	0.386945	1.24408	0.627648	0.0253*	
H1c3	0.510721	0.997491	0.25954	0.0251*	
H1c4	0.419502	1.150888	0.023184	0.0294*	
H1c8	0.11748	1.177084	0.697131	0.026*	
H1c9	0.504952	1.316125	0.497307	0.0297*	
H2c9	0.372858	1.415883	0.504826	0.0297*	
H1c10	0.116351	0.758191	0.705713	0.0286*	
H1c11	0.236448	1.284933	0.063317	0.0283*	
H1c12	0.26825	0.751886	0.605019	0.0266*	
H1c13	0.335867	1.429715	0.368634	0.0288*	
H2c13	0.448556	1.310747	0.361506	0.0288*	
H1c14	0.151711	1.127692	0.438843	0.0247*	
H2c14	0.138427	1.292231	0.450322	0.0247*	
H1c17	0.229709	1.038565	0.326799	0.0253*	
H2c17	0.382737	1.074935	0.36675	0.0253*	
H2	0.1570 (14)	1.2826 (15)	0.2588 (10)	0.0286*	
H1	0.3808 (15)	1.0424 (16)	0.5183 (9)	0.027*	

### Atomic displacement parameters (Å^2^)

**Table:**

	*U*^11^	*U*^22^	*U*^33^	*U*^12^	*U*^13^	*U*^23^
F1	0.0325 (4)	0.0281 (4)	0.0293 (4)	0.0037 (3)	0.0133 (3)	−0.0034 (3)
F2	0.0408 (4)	0.0331 (5)	0.0338 (4)	0.0006 (3)	0.0235 (3)	0.0031 (3)
O1	0.0263 (4)	0.0211 (5)	0.0221 (4)	0.0031 (3)	0.0096 (3)	−0.0006 (3)
O2	0.0268 (4)	0.0238 (5)	0.0209 (4)	0.0045 (3)	0.0038 (3)	0.0029 (3)
N1	0.0247 (5)	0.0200 (5)	0.0177 (4)	0.0002 (4)	0.0061 (4)	0.0010 (4)
N2	0.0211 (4)	0.0196 (5)	0.0177 (5)	−0.0005 (4)	0.0045 (3)	0.0005 (3)
C1	0.0263 (5)	0.0200 (6)	0.0173 (5)	−0.0004 (4)	0.0045 (4)	−0.0016 (4)
C2	0.0231 (5)	0.0168 (6)	0.0207 (5)	−0.0026 (4)	0.0027 (4)	−0.0006 (4)
C3	0.0240 (5)	0.0170 (6)	0.0214 (6)	−0.0024 (4)	0.0025 (4)	−0.0008 (4)
C4	0.0313 (6)	0.0247 (6)	0.0183 (5)	−0.0058 (5)	0.0067 (4)	−0.0015 (4)
C5	0.0234 (5)	0.0172 (6)	0.0175 (5)	−0.0027 (4)	0.0028 (4)	−0.0008 (4)
C6	0.0191 (5)	0.0235 (6)	0.0155 (5)	0.0010 (4)	0.0011 (4)	−0.0003 (4)
C7	0.0210 (5)	0.0207 (6)	0.0143 (5)	0.0003 (4)	0.0011 (4)	−0.0007 (4)
C8	0.0250 (5)	0.0221 (6)	0.0183 (5)	0.0029 (5)	0.0046 (4)	−0.0010 (4)
C9	0.0281 (6)	0.0228 (6)	0.0242 (6)	−0.0056 (5)	0.0063 (4)	0.0009 (4)
C10	0.0266 (6)	0.0227 (6)	0.0222 (6)	−0.0022 (5)	0.0038 (4)	0.0035 (4)
C11	0.0292 (6)	0.0215 (6)	0.0193 (5)	−0.0027 (5)	0.0015 (4)	0.0028 (4)
C12	0.0261 (6)	0.0199 (6)	0.0201 (5)	0.0020 (4)	0.0022 (4)	−0.0014 (4)
C13	0.0290 (6)	0.0206 (6)	0.0236 (6)	−0.0023 (5)	0.0081 (4)	0.0017 (4)
C14	0.0212 (5)	0.0221 (6)	0.0189 (5)	0.0012 (4)	0.0044 (4)	0.0013 (4)
C15	0.0248 (5)	0.0190 (6)	0.0244 (6)	−0.0028 (4)	0.0075 (4)	−0.0042 (4)
C16	0.0245 (5)	0.0295 (7)	0.0199 (5)	−0.0006 (5)	0.0081 (4)	0.0005 (5)
C17	0.0261 (5)	0.0198 (6)	0.0177 (5)	0.0020 (4)	0.0047 (4)	0.0025 (4)

### Geometric parameters (Å, º)

**Table:**

F1—C15	1.3706 (14)	C4—H1c4	0.96
F2—C16	1.3648 (15)	C5—C17	1.5116 (16)
O1—C6	1.3680 (14)	C6—C7	1.4034 (16)
O1—H1	0.937 (16)	C6—C12	1.3879 (16)
O2—C2	1.3628 (14)	C7—C8	1.3962 (16)
O2—H2	0.903 (16)	C8—C16	1.3780 (17)
N1—C13	1.4919 (14)	C8—H1c8	0.96
N1—C14	1.4581 (15)	C9—C13	1.5348 (15)
N1—C17	1.4867 (15)	C9—H1c9	0.96
N2—C1	1.4694 (14)	C9—H2c9	0.96
N2—C9	1.4689 (15)	C10—C12	1.3916 (17)
N2—C14	1.4741 (13)	C10—C16	1.3750 (17)
C1—C7	1.5089 (16)	C10—H1c10	0.96
C1—H1c1	0.96	C11—H1c11	0.96
C1—H2c1	0.96	C12—H1c12	0.96
C2—C5	1.4070 (14)	C13—H1c13	0.96
C2—C11	1.3918 (16)	C13—H2c13	0.96
C3—C5	1.3928 (16)	C14—H1c14	0.96
C3—C15	1.3761 (16)	C14—H2c14	0.96
C3—H1c3	0.96	C17—H1c17	0.96
C4—C11	1.3902 (17)	C17—H2c17	0.96
C4—C15	1.3789 (15)		
			
C6—O1—H1	102.5 (10)	N2—C9—H2c9	109.47
C2—O2—H2	104.6 (9)	C13—C9—H1c9	109.47
C13—N1—C14	103.64 (8)	C13—C9—H2c9	109.47
C13—N1—C17	111.71 (9)	H1c9—C9—H2c9	114.55
C14—N1—C17	111.82 (9)	C12—C10—C16	118.04 (11)
C1—N2—C9	112.60 (8)	C12—C10—H1c10	120.98
C1—N2—C14	115.28 (9)	C16—C10—H1c10	120.98
C9—N2—C14	102.96 (8)	C2—C11—C4	120.53 (10)
N2—C1—C7	112.49 (9)	C2—C11—H1c11	119.73
N2—C1—H1c1	109.47	C4—C11—H1c11	119.73
N2—C1—H2c1	109.47	C6—C12—C10	120.64 (11)
C7—C1—H1c1	109.47	C6—C12—H1c12	119.68
C7—C1—H2c1	109.47	C10—C12—H1c12	119.68
H1c1—C1—H2c1	106.28	N1—C13—C9	105.99 (9)
O2—C2—C5	121.43 (10)	N1—C13—H1c13	109.47
O2—C2—C11	118.03 (9)	N1—C13—H2c13	109.47
C5—C2—C11	120.53 (10)	C9—C13—H1c13	109.47
C5—C3—C15	119.60 (10)	C9—C13—H2c13	109.47
C5—C3—H1c3	120.2	H1c13—C13—H2c13	112.74
C15—C3—H1c3	120.2	N1—C14—N2	103.95 (8)
C11—C4—C15	117.95 (11)	N1—C14—H1c14	109.47
C11—C4—H1c4	121.03	N1—C14—H2c14	109.47
C15—C4—H1c4	121.03	N2—C14—H1c14	109.47
C2—C5—C3	118.46 (10)	N2—C14—H2c14	109.47
C2—C5—C17	121.24 (10)	H1c14—C14—H2c14	114.48
C3—C5—C17	120.20 (9)	F1—C15—C3	118.37 (9)
O1—C6—C7	121.16 (10)	F1—C15—C4	118.77 (10)
O1—C6—C12	118.35 (10)	C3—C15—C4	122.85 (11)
C7—C6—C12	120.48 (10)	F2—C16—C8	118.27 (11)
C1—C7—C6	121.21 (10)	F2—C16—C10	118.96 (11)
C1—C7—C8	120.01 (10)	C8—C16—C10	122.77 (11)
C6—C7—C8	118.62 (10)	N1—C17—C5	111.70 (9)
C7—C8—C16	119.41 (11)	N1—C17—H1c17	109.47
C7—C8—H1c8	120.29	N1—C17—H2c17	109.47
C16—C8—H1c8	120.29	C5—C17—H1c17	109.47
N2—C9—C13	103.87 (9)	C5—C17—H2c17	109.47
N2—C9—H1c9	109.47	H1c17—C17—H2c17	107.14

### Hydrogen-bond geometry (Å, º)

**Table:**

*D*—H···*A*	*D*—H	H···*A*	*D*···*A*	*D*—H···*A*
O1—H1···N2	0.937 (16)	1.756 (16)	2.6413 (12)	156.2 (14)
O2—H2···N1	0.903 (16)	1.821 (15)	2.6579 (12)	153.2 (13)
C11—H1*c*11···O1^i^	0.96	2.44	3.4001 (13)	174.43
C17—H2*c*17···O1^ii^	0.96	2.55	3.4837 (13)	166

Symmetry codes: (i) −*x*+1/2, *y*+1/2, −*z*+1/2; (ii) −*x*+1, −*y*+2, −*z*+1.
